# Twice weekly dosing with Sebelipase alfa (Kanuma®) rescues severely ill infants with Wolman disease

**DOI:** 10.1186/s13023-024-03219-5

**Published:** 2024-06-25

**Authors:** María José de Castro, Simon A Jones, Javier de las Heras, Paula Sánchez-Pintos, María L Couce, Cristóbal Colón, Pablo Crujeiras, María Unceta, Heather Church, Kathryn Brammeier, Wu Hoi Yee, James Cooper, Laura López de Frutos, Irene Serrano-Gonzalo, María José Camba, Fiona J. White, Victoria Holmes, Arunabha Ghosh

**Affiliations:** 1 Unit of Diagnosis and Treatment of Congenital Metabolic Diseases, Department of Neonatology, Santiago de Compostela University Clinical Hospital, Santiago de Compostela, Spain; 2 Centro de Investigación Biomédica en Red de Enfermedades Raras (CIBERER), European Reference Network for Hereditary Metabolic Disorders (MetabERN), IDIS-Health Research Institute of Santiago de Compostela, Santiago de Compostela University Clinical Hospital, Santiago de Compostela, Spain; 3 Willink Biochemical Genetics Unit, St Mary’s Hospital, Manchester University Foundation Trust, University of Manchester, Manchester, ZIP M13 9WL UK; 4 School of Biological Sciences, Facutly of Biology, Medicine and Health, The University of Manchester, Manchester, UK; 5 Division of Pediatric Metabolism at Cruces University Hospital, Centro de Investigación Biomédica en Red de Enfermedades Raras (CIBERER), European Reference Network for Hereditary Metabolic Disorders (MetabERN), Biocruces-Bizkaia Health Research Institute, University of the Basque Country (UPV/EHU), Barakaldo, Spain; 6 Fundación Española para el Estudio y Terapéutica de la Enfermedad de Gaucher y otras lisosomales (FEETEG), Zaragoza, Spain

## Abstract

**Background:**

Sebelipase alfa (Kanuma®) is approved for patients with Wolman disease (WD) at a dosage of 3–5 mg/kg once weekly. Survival rates in the second of two clinical trials was greater, despite recruiting more severely ill patients, probably related to higher initial and maximal doses. We aimed to evaluate the effective pharmacokinetics and pharmacodynamics of Sebelipase alfa when administered to patients with severe WD at 5 mg/kg twice weekly, an intensive regimen which was not assessed in the trials.

**Methods:**

We recruited 3 patients receiving Sebelipase alfa 5 mg/kg twice weekly. We measured LAL activity in leukocytes and plasma oxysterol concentration in two patients and LAL activity in fibroblasts in one patient. Clinical follow up was also assessed.

**Results:**

Analyses of LAL activity and oxysterols demonstrate that there is short-lived enzyme activity post-dosing which is associated with the release of stored lipids. Clinical data demonstrate that 5 mg/kg twice weekly dosing is well tolerated and effective.

**Conclusion:**

5 mg/kg twice weekly dosing with Sebelipase alfa rescues severely ill infants with WD by increasing substrate clearance. There is biologically relevant lipid accumulation in the ‘trough’ periods before the next dosing, even with this intensive regimen.

## Introduction

Lysosomal acid lipase deficiency (LAL-D) is a rare inherited autosomal recessive metabolic disease with an estimated prevalence of 0,2 per 10,000 individuals in Europe [1, 2]. LAL-D is caused by pathogenic variants in the *LIPA* gene (MIM*613,497), which encodes an essential enzyme in the metabolism and degradation of cholesteryl esters and triglycerides (EC:3.1.1.13) [3, 4]. Absence or decreased levels of LAL leads to the accumulation of the aforementioned substrates mainly in the liver, spleen, and small intestines [5, 6].

Wolman disease (WD) is the most severe form of LAL-D and is characterized by early onset and rapid progression. Clinical manifestations include failure to thrive, vomiting, diarrhoea, hepatosplenomegaly [7] and calcification of the adrenal glands [8]. Another common initial presentation is with a secondary hemophagocytic syndrome [9–11]. Without treatment, infants with WD do not survive beyond 6 months of age [12]. Diagnosis is based on clinical symptoms, measurement of LAL enzyme activity [13], and molecular genetic confirmation [14].

Sebelipase alfa (Kanuma®, Kanuma™) has been approved since 2015 as a long-term enzyme replacement therapy (ERT) for patients diagnosed with LAL-D [15–17]. Sebelipase alfa binds to the mannose 6-phosphate receptor and the macrophage mannose receptor and is subsequently internalized into the lysosome, catalysing the hydrolysis of cholesteryl esters and triglycerides [18].

The efficacy, safety and pharmacokinetics of Sebelipase alfa in patients with WD were evaluated in two pivotal multicentre phase II/III studies: LAL-CL03 (VITAL) [19] and LAL-CL08[20]. The VITAL study included 9 patients who presented with growth retardation, severe hepatosplenomegaly and rapidly progressive liver disease before the age of 6 months. All patients received weekly intravenous (IV) infusions of Sebelipase alfa at a starting dose of 0.35 mg/kg (8 patients) or 0.20 mg/kg (1 patient) for at least 2 infusions, followed by a weekly dose of 1 mg/kg or 3 mg/kg based on clinical response. LAL-CL08 included 10 patients with WD which appeared to have more severe disease at baseline. Sebelipase alfa treatment was started at a higher dose, and faster dose escalation was permitted. All participants started treatment with weekly Sebelipase alfa infusions at a dose of 1 mg/kg and they could be considered for a dose increase to 3 mg/kg and up to 5 mg/kg. Notably, survival was better overall in the LAL-CL08 study, with Kaplan-Meier estimates of survival being 67% (to 12 months) and 56% (to 4 years) in VITAL, compared to 90% (to 12 months) and 80% (to 3 years) in CL08. This increased survival in the CL08 study compared to VITAL may be related to the higher initial and maximal doses of ERT, with faster dose escalation [20] .

Following these pivotal trials, Sebelipase alfa received marketing authorisation at a licensed dose of 3 mg /kg weekly for WD. Recently, the data sheet has been updated to recommend the administration of higher doses (5 mg/kg weekly) when a suboptimal clinical response is observed.

This article postulates that patients with more advanced or rapidly progressing WD may benefit from intensification of the ERT regimen. We carried out in vivo and in vitro pharmacodynamic evaluations in three patients with WD and multiple organ failure who were rescued with an intensive regimen of ERT with Sebelipase alfa at 5 mg/kg twice weekly, describing safety, efficacy, and clinical follow up.

## Materials and methods

Written consent for publication of patient data was obtained from the families of all individuals included. Demographic information and clinical data was collected from medical records, and haematological and biochemical data was obtained from hospital laboratory records.

### LAL enzyme activity

LAL activity was measured in mixed leucocytes in patients 1 and 2 using the artificial substrate 4-methylumbelliferyl-palmitate (NBS Biologicals, UK) based on the previously published protocol [21,22]. This assay has also been used to measure LAL activity in cultured skin fibroblasts as part of a pulse-chase experiment with Sebelipase alfa in patient 3.

### Molecular genetic diagnosis

In patients 1, 2 and 3 molecular genetic analysis was performed by Sanger sequencing. All exons and intron-boundaries, as well as 3’ and 5’-UTR region for *LIPA* gene were amplified by homemade design PCR. Products were sequenced by capillary electrophoresis using a SeqStudio analyser (Applied Biosystems), and results were compared with the GeneBank database’s reference sequence (NG_008194.1).

### Plasma oxysterols

In patients 1 and 2, serial measurements of plasma oxysterol concentrations were used as a surrogate disease biomarker. 7-ketocholesterol (7-KC) was quantified by liquid chromatography and coupled tandem mass spectrometry as previously reported [23–25].

### Cell culture

Cultured skin fibroblasts from patient 3 were used as an in-vitro model for enzyme uptake and clearance. Cells are routinely cultured in complete Minimum Essential Medium (MEM), 10% FCS, 5 mM glutamine, non-essential amino acids (Gibco, UK) in 5% carbon dioxide.

Enzyme challenge - Cells were plated into T12.5 flasks x 10 and grown to ~ 95% confluence. Each flask (minus one baseline control) was challenged with sebelipase alpha at a concentration of 100 ng/ml diluted in complete MEM (500 ng in 5 ml complete MEM per T25 flask) and incubated for 1 h at 37 oC in 5% carbon dioxide. Following exposure, the sebelipase alfa conditioned medium was removed, and fresh 5 ml complete medium was replaced into each flask. The cells were then harvested with trypsin, washed in isotonic saline, and then stored at -80oC ready for enzyme analysis at successive time points: time zero, 1, 2, 4,6 ,24, 48, 72, 96 and 120 h post-sebelipase alfa challenge. For a baseline control a single flask was harvested at time zero without exposure to sebelipase alfa as a baseline diagnostic measurement for this patient cell line. The activity of LAL within the harvested fibroblasts was then measured using the above protocol [21].

## Results

### Demographics, safety and clinical follow up in severely ill WD patients receiving sebelipase alfa 5 mg/kg twice weekly

#### Patient 1

Patient 1 presented aged 3.5 months with fever, pancytopenia, hepatosplenomegaly and associated hypertriglyceridemia (max. value 1,950 mg/dL), hyperferritinemia (max. value 11,300 ng/mL) and transaminitis (max. values AST 177 UI/L, ALT 106 UI/L, GGT 266 UI/L). A diagnosis of hemophagocytic lymphohistiocytosis was made, and she was initially treated with steroids and etoposide. Due to poor evolution and lack of response to treatment, further investigations confirmed a severe phenotype of WD. LAL enzymatic activity quantified on DBS was 0.04 nmol/punch/h (normal range 0.71–2.38 nmol/punch/h). 7-KC at diagnosis was 2313 ng/mL (normal range: 2-104 ng/mL). Genetic testing identified the NM_000235.2:c.966 + 2T > G variant in a homozygous state. This variant was previously described in a Spanish WD cohort with a lethal outcome [26]. Patient 1 was transferred to the paediatric intensive care unit due to respiratory distress, with massive hepatosplenomegaly reaching the iliac fossa, alongside severe thrombocytopenia and neutropenia. The first infusion of Sebelipase alfa was administered one day after the diagnosis, at a dose of 3 mg/kg. She deteriorated rapidly during the following days, developing acute respiratory failure that required intubation and mechanical ventilation, as well as acute renal failure that required the initiation of haemodiafiltration. After confirming adequate tolerance to the first infusion of sebelipase and given the acutely life-threatening situation of the patient (pulmonary oedema, acute renal failure, ascites, thrombocytopenia with transfusion dependence and hyper-inflammatory state), it was decided to increase the dose of ERT to 5 mg/kg (infused over 4 h) and administer it twice weekly. The patient’s overall clinical status improved during the following weeks, with mechanical ventilation being discontinued after 2.5 weeks, and haemodiafiltration discontinued after 3 weeks. A progressive improvement in the analytical parameters related to inflammation, with a marked decrease in ferritin and triglyceride levels during the first week of the intensified ERT regimen, as well as resolution of leukopenia. Hemoglobin levels and platelet counts improved from the second week on, although the patient continued to require daily blood product transfusions during the first month of hospitalization, which subsequently reduced in frequency and stopped by 2 months. Regarding lysosomal disease biomarkers, during the first week CCL18/PARC and ChT decreased (35 ng/mL and 138 nmol/mL/h respectively), but 7-KC was still rising (2944 ng/mL). Three weeks after the diagnosis, 7-KC began to decrease (398 ng/mL). During the first 15 days of admission and due to the clinical instability of the patient, she was maintained with total parenteral nutrition without lipids. Subsequently, enteral nutrition was started with a protein hydrolysate and lactose free modular formula (carbohydrate intake: 18 mg/kg/min, protein intake: 4 g/kg/day and lipid intake: 1.0–1.5 g/kg/day, with a total caloric intake of 120 to 140 Kcal/day). Weight was static during the first two months of admission, but improved thereafter (5 kg at 4 months of age – corresponding to percentile 10 – on the first day of admission, and 7 kg at 6 months of age – corresponding to percentile 35 - prior to discharge). Finally, a clinically meaningful decrease in the size of the liver and spleen was observed.

Patient 1 remains well on 5 mg/kg once weekly ERT, stepped down after 3 months, which was well tolerated, with no anaphylaxis or adverse reactions

### Patient 2

The patient, a full-term male newborn with normal birth weight (3.23 kg), presented with vomiting and abdominal distension from the first days of life, and was admitted in hospital at 6 weeks of age due to failure to thrive. He was pale, and abdominal distension with splenomegaly and cachexia were evident, with a body weight of 3.52 kg, below the 3rd percentile. Laboratory investigations showed HDL < 5 mg/dL, raised transaminases (AST: 225 IU/L, ALT: 237 IU/L, GGT: 157 IU/L), high ferritin (1072 ng/mL), and low fat soluble vitamins (25-OH-vitamin D: 13 ng/mL, vitamin A: 0.08 mg/L and vitamin E: 1.3 mg/L). Blood film examination revealed vacuolated lymphocytes and 7-KC levels were increased (906 ng/mL; normal range 2 to 103.6). LAL-D diagnosis was confirmed after the determination of LAL enzyme activity in DBS (0.02 nmol/punch/h) and genetic testing, which revealed the NM_000235.2:c.966 + 2T > G variant in a homozygous state. The patient started enteral modular feeds containing glucose polymer, medium-chain fat and an amino acid mixture at 1 month and 23 days of age, and a day later ERT with Sebelipase at 5 mg/kg/week was started. Due to the intestinal damage, there was a malabsorption worsening with a weight loss of 250 g in 24 h, and TPN was started. At that moment, the decision was made to increase Sebelipase dosage up to 5 mg/kg/twice a week. This twice a week infusion regimen was maintained for 4 weeks. At 3 months of age TPN was stopped, and the patient was discharged home eight days later. At the time of writing, the patient is almost 5 months, with a body weight of 6.75 kg (p25).

## Patient 3

Patient 3 presented at the age of 2.5 months with a typical history for WD, including vomiting and loose stools since birth, hepatosplenomegaly and ascites, with lipid storage in Kupffer cells on liver biopsy. The diagnosis was confirmed by both enzyme analysis (leukocyte acid esterase activity 32 nmol/mg/hr, reference range 35 − 2,000 nmol/mg/h) and molecular genetics, which identified a homozygous whole gene deletion of *LIPA*, previously reported in a series of patients with WD of South Asian background [27]. Enzyme replacement therapy with sebelipase alfa at an initial dose of 5 mg/kg weekly was commenced at 3 months of age, and he was changed from a high energy feed with significant fat content (6.3 g total fat/kg/d, 60% MCT) to fat-free total parenteral nutrition. At baseline, he had hypoalbuminaemia (26 g/L), mildly elevated triglycerides (2.7 mmol/L) and C-reactive protein (32 mg/L). He was anemic (hemoglobin 76 g/L), but without thrombocytopenia or leukopenia. Transaminases and ferritin were normal. Plasma oxysterols (cholestane-3β, 5α, 6β-triol) at baseline were elevated at 107 ng/ml (reference range 10–37 ng/ml). During the first 2–3 weeks on ERT, a hyper-inflammatory picture became apparent, with persistently raised CRP, intermittent fever without positive blood cultures, and a requirement for repeated blood transfusions. Abdominal distension and ascites markedly increased, associated with rapid weight gain and tachypnoea, and this persisted despite treatment with fluid restriction and diuretics (furosemide and spironolactone). Due to ongoing respiratory distress, he was admitted to the paediatric intensive care unit after 5 weeks on ERT, and commenced non-invasive ventilatory support. There was a modest improvement in tachypnoea following needle aspiration of ascitic fluid, but this was not sustained. Given the hyperinflammatory picture, ascites and respiratory distress, the frequency of enzyme replacement therapy was increased (to 5 mg/kg twice weekly). Following this, he made a good recovery and was stepped down from intensive care after one week. There was clinical and radiological improvement in ascites with only a trace of free fluid observable on ultrasound after 6 months on ERT. By this point, weight gain had stabilised, tachypnoea and fever had resolved, he was no longer transfusion dependent, and he had established enteral feeding with an amino acid based, high carbohydrate, minimal fat (0.8 g LCT for essential fatty acids) modular feed. Organomegaly improved, with 6 cm hepatomegaly and no palpable splenomegaly. Plasma oxysterols reduced to below the upper limit of normal within one month of starting twice weekly ERT, and remain within the reference range. Anti-sebelipase antibody titres peaked at a moderate titre of 1:16384 (7 weeks after commencing ERT), and have since remained stable. Overall, his clinical condition is vastly improved and he was discharged home after a total inpatient stay of 6 months. After 2 months he was stepped down to once weekly sebelipase at 5 mg/kg.

## Effective pharmacokinetics in patients 1 and 2

**Fig. 1:**
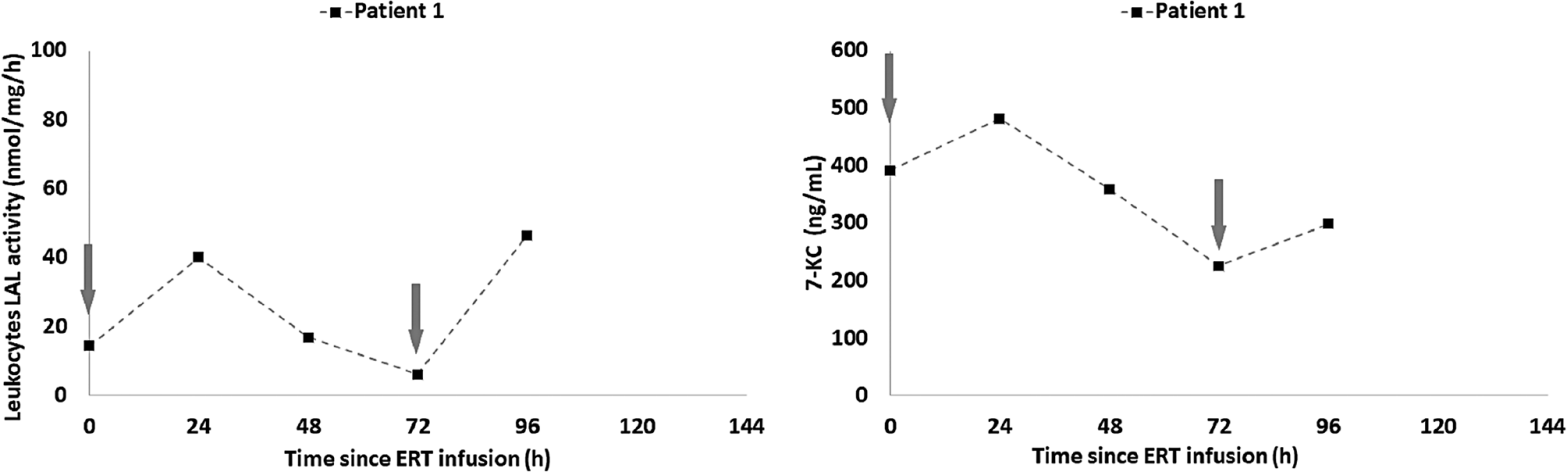
Patient 1 LAL activity levels and 7-KC concentration after 1.5 month receiving sebelipase at a dose of 5 mg/kg twice weekly

**Fig. 2:**
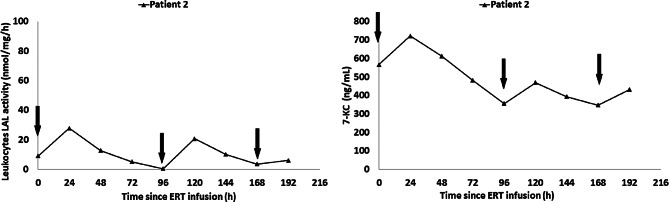
Patient 2 LAL activity levels and 7-KC concentration after 1.5 month receiving sebelipase at a dose of 5 mg/kg twice weekly

We carried out serial determinations of LAL activity in leukocytes and quantification of plasma oxysterols (7-KC), to explore the pharmacokinetics and pharmacodynamics of this intensive regimen. Six time points were included: pre-infusion before the first of two weekly doses of sebelipase alfa (0 h); 24 h after the first weekly dose of Sebelipase alfa (24 h); 72 h after the first weekly dose of Sebelipase alfa (72 h); 96 h after the first weekly dose of sebelipase alfa, which coincides with the pre-infusion time point of the second weekly dose of sebelipase alfa (96 h); 120 h after the first weekly dose of sebelipase alfa which coincides with 24 h after the second weekly dose of sebelipase alfa (120 h); and 144 h after the first weekly dose of sebelipase alfa which coincides with 48 h after the second weekly dose of sebelipase alfa (144 h). Results corresponding to patient 1 are displayed in Fig. 1. It must be noted that due to worsening anaemia, the volume of blood obtained at the 144 h time point was only sufficient to analyse 7-KC concentrations. Results are displayed in Fig. 1 for patient 1 and Fig. 2 for patient 2.

As shown in Fig. 1, the baseline level of LAL activity was abnormally low (15 nmol/mg/h; normal range: 258–1141 nmol/mg/h), similar to levels observed in untreated WD patients. Twenty-four hours after the first twice weekly dose of Sebelipase alfa, a rapid increase in LAL activity was observed up to 40 nmol/mg/hour, corresponding to an increase of 133% of the basal activity. Subsequently, a rapid decrease in LAL activity was observed, reaching baseline values within 72 h after. Similarly, 24 h after the second weekly dose of sebelipase alfa, there is evidence of a rapid rise in LAL activity up to 50 nmol/mg/h.

In parallel, two peaks of 7-KC in plasma (498 ng/mL and 300 ng/mL) are observed at 24 h after the first and the second twice weekly doses of Sebelipase alfa respectively, and progressively decreasing between infusions, showing a double clearance of substrate. Baseline 7-KC concentration prior to the first twice weekly dose of Sebelipase alfa was 400 ng/mL (normal range: 2–104 ng/mL) and fell to 225 ng/mL prior to the second weekly dose of Sebelipase alfa. However, despite the aforementioned double clearance mechanism, oxysterol levels in plasma remain significantly high with values compatible with the diagnosis of WD and did not normalize, even after receiving the intensive regimen of ERT for 1.5 months.

As shown in Fig. 2, the baseline level of LAL activity was abnormally low (9.20 nmol/mg/h; normal range: 258–1141 nmol/mg/h). Twenty-four hours after the first twice weekly dose of Sebelipase alfa, a rapid increase in LAL activity was observed, up to 28 nmol/mg/hour. Subsequently, a rapid decrease in LAL activity was observed. Similarly, 24 h after the second weekly dose of sebelipase alfa, there is evidence of a rapid rise in LAL activity up to 20.8 nmol/mg/h.

### Effective pharmacokinetics in fibroblasts (patient 3)

Serial LAL activity was measured in cultured fibroblasts obtained from a historical WD patient as part of a pulse-chase experiment. Addition of Sebelipase showed a significant peak above physiological range initially but this had decreased to almost baseline values by 48–72 h post infusion. Fibroblasts probably take up lysosomal enzymes via the M-6P receptor whereas macrophages predominantly use the mannose receptor. Importantly, both cell types in vivo and in vitro consistently show a relatively short intracellular half life.

**Fig. 3:**
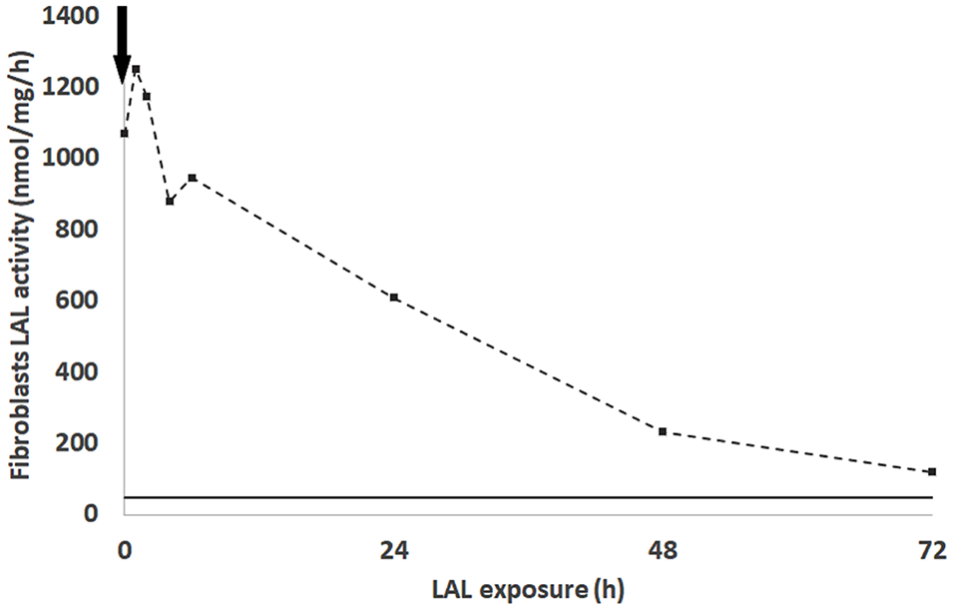
Patient 3 fibroblast clearance of Sebelipase. Black arrows denote the time of Sebelipase addition. The solid black line shows the activity of the same patient fibroblast before the treatment

## Discussion

WD is a rare, progressive infantile onset disease caused by insufficient LAL activity and leading to death with a median age of 3.5 months [12]. The disease hallmarks include liver failure, hepatosplenomegaly, steatorrhea and malabsorption, and adrenal calcification due to extensive storage of cholesteryl esters and triglycerides in the lysosomes of Kupffer cells, hepatocytes and macrophages [28]. Sebelipase alfa is licensed for long-term treatment of infants with WD after receiving marketing authorization from the Food and Drug Administration and the European Medicine Agency in 2015, following an accelerated assessment due to unmet medical need [29].

Due to the very low incidence of WD, the number of participants in the pivotal clinical trials of sebelipase alfa was necessarily small. The collection of long-term efficacy data is therefore mandatory. In the clinical trials VITAL (*N* = 9) and CL08 (*N* = 10), significant improvements were observed for a number of disease parameters and there was an improvement in survival of infants with WD. This survival benefit appeared to be even greater in the second clinical trial, CL08, which allowed for higher starting doses, faster dose escalation, and higher maximal doses, as compared to the VITAL study [20].

Dose-dependent responses to recombinant human LAL have been demonstrated in a mouse model of LAL-D. However, the *LIPA*^*-/-*^ mouse is not an ideal model for WD as it resembles the later onset phenotype more closely [30, 31]. It also does not demonstrate the inflammatory disease seen so frequently in infants. Two comparative preclinical studies with recombinant human LAL in *LIPA*^-/-^ mice [32] were conducted with lower doses (0.8 and 3.2 mg/kg weekly), beginning at 16 weeks (study 1) and with higher dose (10 mg/kg) in early (8 weeks), middle (16 weeks) and late (24 weeks) disease stage (study 2). Systemic evaluation of the dosage and treatment initiation age on lipids, organ size, body weight and life span in *LIPA*^-/-^ mice revealed that treatment started at early or middle disease stages resulted in greater effects than later stage initiations, and survival was also dose dependent (in study 1 ERT extended the life span by 52 days with 0.8 mg/kg versus 94 days with 3.2 mg/kg). Also, the higher dose of ERT (10 mg/kg) given weekly was able to prevent disease progression and even reversal of advanced disease. These studies revealed some issues that remain relevant to the dosing of ERT for patients with WD, especially those more severely affected with advanced disease. This includes the potential need for dose variation depending upon disease stage, and raises the possibility of reversal of disease manifestations with higher dosing, even in late stage disease.

Higher doses than 5 mg/kg weekly were not tested in the clinical trials due to a prior assumption that the optimal dose would be between 1 and 3 mg/kg weekly (of note the licensed dose for late onset LAL-D is 1 mg/kg alternate weekly). More than once weekly dosing was not tested in either the pre-clinical model or human trials. However, there are previous data from three other pre-clinical studies employing recombinant human LAL produced in different systems. In these studies, using a 3-day interval of administration, the authors showed normalization of hepatic color, decreases in hepatic cholesterol and triglycerides contents, and diminished foamy macrophages in liver, spleen and intestinal villi in a dose dependent manner [33–35]. They also demonstrated that the half-life of the recombinant enzyme was 14 h and 32 h in liver and spleen, respectively [33].

Pharmacokinetics (PK) of enzyme replacement therapies are traditionally evaluated as plasma levels. These levels however are rarely informative for dosing decisions as lysosomal enzymes are usually only active at lysosomal pH. More useful information may be gained from understanding the intracellular PK over time and following an intravenous infusion. While this can be challenging in LSDs with multi-organ storage, in WD the macrophage is the main storage cell. Using a mixed leukocyte preparation permits accurate understanding of active enzyme in the relevant cell type in this disease. We have termed this the ‘effective PK’. Our LAL activity data, both in leukocytes and fibroblasts, support the idea that there is short-lived enzyme activity post-dose, suggesting that sebelipase alfa is present in cells for only 2–3 days post-administration. When combined with plasma oxysterol levels, it is shown that the increase in LAL activity after twice weekly ERT administration is biologically important, as it is associated with a temporary rise in oxysterol, presumably related to release of previously stored lipids and partially oxidised cholesterol, exerting a further clearance of substrate. Importantly, the fact that oxysterol levels were persistently high in patient 1, without normalization even after 1.5 months of twice weekly sebelipase, shows that there is still biologically relevant lipid storage in the ‘trough’ periods before the next dosing. This may suggest that even with twice weekly infusions, no steady state is truly achieved. Correspondingly, samples taken intermittently in patient 3 over the course of several weeks, showed higher leukocyte LAL activity in all inter-infusion samples compared to all pre-infusion ‘trough’ samples (supplemental Fig. 1). This may be of special interest in the most severely ill patients presenting in extremis, with a potential for increased chance of survival by correcting the pathophysiology of the disease more quickly and avoiding the rapid progression to multi-organ failure.

While the survival benefit of sebelipase alfa was clear from the two clinical trials, even in the second trial some infants died. This was recognised to be often those infants presenting in later stages of disease, with significant inflammation and more severe end organ damage. Individuals described in this report had a severely compromised clinical status, consistent with a more advanced stage of WD. Due to their life-threatening condition and the hypothesis that higher doses of sebelipase may lead to better outcomes, the medical teams decided to administer intensive high dose twice weekly treatment. This was associated with progressive improvement in clinical status and ultimately led to the rescue and survival of the three patients included in this cohort, and without any severe adverse events related to the increased dosage.

One possible concern of the administration of this intensive regimen of sebelipase alfa twice weekly would be the increased risk of hypersensitivity reactions. Nevertheless, all patients were successfully maintained on twice weekly infusions as long as was clinically indicated, despite occasional infusion reactions as previously reported, thus confirming the acceptable safety profile of this therapeutic strategy. This is especially relevant when dealing with unstable patients with an advanced stage of WD, in whom the risk of decompensation may be increased.

We acknowledge limitations to this study including the small sample size, the lack of a head-to-head comparison and no blinding. However, given the rarity and lethality of WD, little is known about an optimal ERT dosing and the post-licensing environment does not allow for a formal clinical trial evaluation.

Our PK approach in this paper differs from the typical model. However, we believe it may offer advantages in lysosomal storage disorders when the drug is typically only active once intracellular. Preliminary work in our laboratory suggests other recombinant enzymes (e.g. laronidase) are much more persistent in leukocytes and reach a steady state with weekly dosing (unpublished observations). This clearly does not occur with Sebelipase and this information is critical to dosing decisions in the most extreme cases. The concept of ‘effective pharmacokinetics’ may be relevant for a number of other ERTs in this area.

The recently published article by Demaret et al. [15] shows a 100% survival rate in a cohort of five patients with WD, compared to a survival rate of 55% and 80% in the LAL-CL03 and LAL-CL08 studies, respectively. The authors attribute these good results to the high proportion of positive family history in their cohort (3/5), leading to an early diagnosis (between 0 and 2 months of age) and better clinical status at ERT initiation. We therefore consider that there is an unmet need for those patients who are diagnosed in more advanced stages of the disease with profoundly compromised clinical status, in which a higher dose increased frequency regimen may be of benefit.

Some authors advocate for multimodal treatment of WD [27], including ERT and dietary substrate reduction diet (SRD) combined with hematopoietic stem cell transplantation (HSCT). ERT and SRD improves the HCT process and HCT likely provides better enzyme delivery to tissues and better long term outcomes, than those achievable with ERT alone. There is a need for a flexible approach to dosing and combination therapy to gain the best outcome for all infants, even those in extremis. This is a good example of precision medicine, optimising the dose and mode of therapy for each individual patient. While accelerated approval for drugs with a dramatic patient benefit like sebelipase alfa are welcome, clinical trials are aimed at regulatory approvals rather than guiding clinical management and designing optimal care pathways. Post-approval clinical trials in such a severe and ultra-orphan disease are unlikely and so case series such as this, supported by opportunistic pharmacokinetic and dynamic measures may be the only way to generate further evidence. While industry registries are common, few have generated major clinically important data [36]. Studies like these remain an important tool for improving outcomes but only if regulators and reimbursement authorities will begin to accept this level of evidence. Specifically for sebelipase alfa, we make the case that a short-term intensification of ERT, with a dose of 5 mg/kg twice weekly, has the potential to rescue even the most severely unwell WD infants who otherwise may not have survived, and we suggest that the approved label could be altered to reflect this new dosing paradigm. The benefits of accelerated approval approaches from regulators will be lessened if there is not also a pragmatic approach to review of post licensing real world data.

## Conclusions

Pharmacokinetics in leukocytes and further in vitro work demonstrates that the intracellular half-life of Sebelipase alfa (Kanuma®) is very short, supporting that twice weekly dosing (which was not tested in the clinical trials) rescues the most unwell children with WD by increasing substrate clearance.

## Data Availability

Data archiving is not mandated but data will be made available on reasonable request.
